# Increased Osteoclast and Decreased Osteoblast Activity Causes Reduced Bone Mineral Density and Quality in Genetic Hypercalciuric Stone‐Forming Rats

**DOI:** 10.1002/jbm4.10350

**Published:** 2020-02-25

**Authors:** Nancy S Krieger, Luojing Chen, Jennifer Becker, Sean DeBoyace, Hongwei Wang, Murray J Favus, David A Bushinsky

**Affiliations:** ^1^ Division of Nephrology University of Rochester School of Medicine Rochester NY USA; ^2^ Section of Endocrinology University of Chicago Pritzker School of Medicine Chicago IL USA

**Keywords:** GENETIC ANIMAL MODELS, OSTEOBLASTS, OSTEOCLASTS, STROMAL/STEM CELLS, DISORDERS OF CALCIUM AND PHOSPHATE METABOLISM

## Abstract

To study human idiopathic hypercalciuria (IH), we developed an animal model, genetic hypercalciuric stone‐forming (GHS) rats, whose pathophysiology parallels that in IH. All GHS rats form kidney stones and have decreased BMD and bone quality compared with the founder Sprague–Dawley (SD) rats. To understand the bone defect, we characterized osteoclast and osteoblast activity in the GHS compared with SD rats. Bone marrow cells were isolated from femurs of GHS and SD rats and cultured to optimize differentiation into osteoclasts or osteoblasts. Osteoclasts were stained for TRAcP (tartrate resistant acid phosphatase), cultured to assess resorptive activity, and analyzed for specific gene expression. Marrow stromal cells or primary neonatal calvarial cells were differentiated to osteoblasts, and osteoblastic gene expression as well as mineralization was analyzed. There was increased osteoclastogenesis and increased resorption pit formation in GHS compared with SD cultures. Osteoclasts had increased expression of *cathepsin K*, *Tracp*, and *MMP9* in cells from GHS compared with SD rats. Osteoblastic gene expression and mineralization was significantly decreased. Thus, alterations in baseline activity of both osteoclasts and osteoblasts in GHS rats, led to decreased BMD and bone quality, perhaps because of their known increase in vitamin D receptors. Better understanding of the role of GHS bone cells in decreased BMD and quality may provide new strategies to mitigate the low BMD and increased fracture risk found in patients with IH. © 2020 The Authors. *JBMR Plus* published by Wiley Periodicals, Inc. on behalf of American Society for Bone and Mineral Research.

## Introduction

Hypercalciuria is the most common metabolic abnormality in patients who form calcium‐ (Ca) containing kidney stones.^1^, ^2^ Elevated urine Ca increases urine supersaturation with respect to the Ca solid phases: Ca oxalate and Ca phosphate. Initial crystal formation occurs in the renal medullary interstitium, which then extends into the urinary space^3^, ^4^ where further crystal growth and aggregation may result in clinically significant stone formation.^2^

The source of the elevated urine Ca appears to be caused by a systemic dysregulation of Ca transport; there is increased intestinal Ca absorption, decreased renal tubule Ca reabsorption, and increased bone resorption.^1^, ^2^, ^5^ Patients with idiopathic hypercalciuria (IH) are often in negative Ca balance as they excrete more Ca in their urine than they absorb from their diet. The negative Ca balance may occur over a broad range of dietary Ca intakes, but is most apparent when dietary Ca intake is low.^6^

Negative Ca balance with persistent hypercalciuria predicts a decrease in BMD in IH patients^7^ that has been borne out by reports of low BMD at the lumbar spine, proximal femur, and/or forearm.^7^, ^8^, ^9^, ^10^ The low BMD contributes to the increased rates of vertebral and hip fracture risk among stone formers.^9^, ^10^, ^11^ Further, the Third National Health and Nutrition Examination Survey (NHANESIII) found that men with a history of nephrolithiasis were more likely to have low femoral neck BMD with lower Ca intake.^12^ BMD has been reported to be inversely correlated with the level of urine Ca excretion in both men and women.^13^ Histomorphometric analyses of bone biopsy specimens suggest that the low bone density in IH patients results from increased bone resorption alone or is accompanied by low bone formation and decreased bone mineralization.^14^, ^15^, ^16^, ^17^ Histologic evidence of increased bone resorption is supported by elevated circulating cytokines and bone resorption turnover markers, Ca kinetic studies, and bone tissue RANKL overexpression.^18^, ^19^, ^20^ However, other studies have shown elevation of some bone turnover biomarkers but not others,^13^, ^20^ and Ca kinetic studies have demonstrated both increased bone resorption and formation.^21^

The inconsistency of bone turnover markers and histomorphometry findings across clinical studies suggest heterogeneity in the pathogenesis of bone disease in IH. However, variation in genetic, nutritional, and endocrine factors can modulate bone cell function and it is difficult to control for these factors in humans. In addition, no direct measurements of bone cellular function or gene expression related to bone formation and resorption have been reported for human IH.

To determine the cellular and molecular basis for alterations in bone formation and resorption in hypercalciuria while controlling for genetic, nutritional, and hormonal factors, we conducted studies in rats from an inbred colony of hypercalciuric rats.^22^ These genetic hypercalciuric stone‐forming (GHS) rats are the result of over 100 generations of selective inbreeding of hypercalciuric offspring of the original spontaneously hypercalciuric male and female Sprague–Dawley (SD) rats. GHS rats share similar patterns of Ca metabolism with IH patients including increased intestinal Ca transport,^23^ decreased renal tubule Ca reabsorption,^24^ and negative Ca balance on a restricted Ca intake,^25^ as well as increased bone resorption.^26^ On a normal Ca diet, all GHS rats form kidney stones and have normal serum Ca and PTH levels.^22^ Serum 1,25(OH)_2_D levels are elevated in 50% to 70% of IH patients^27^ and are normal, not elevated in GHS rats.^22^ We have found that GHS rats have a pathologic overexpression of the vitamin D receptor (VDR) in intestine, kidney, and bone cells, which results in changes in Ca transport in these organs^28^, ^29^, ^30^ through overexpression of numerous VDR‐dependent genes^31^, ^32^, ^33^, ^34^, ^35^ contributing to the hypercalciuria.

The GHS rats have decreased bone density compared with the parental SD rats, including reduced trabecular volume and thickness, and increased brittleness and propensity to fracture^36^, ^37^ that can be at least partially reversed with the thiazide diuretic chlorthalidone.^38^ In the present study, we used GHS and SD rat bone cells to test the hypothesis that net bone resorption is increased in GHS rats by examining the functional status of marrow osteoclast lineage cells and osteoblasts derived from bone marrow stromal cells (BMSCs) and neonatal rat calvariae. The results provide a direct measure of bone cell activity in GHS rats, which suggests an uncoupled turnover with increased resorption and decreased formation leading to low bone density and increased bone fragility.

## Materials and Methods

### Establishment of GHS rats

Adult Sprague–Dawley (SD) rats (Charles River Laboratories, Kingston, NY, USA) were initially screened for hypercalciuria by placing the rats in individual metabolic cages, feeding them a constant amount of a standard (1.2% Ca) Ca diet and measuring urine Ca excretion. The most hypercalciuric male and female rats were used to breed the next generation. A similar protocol was used for screening and inbreeding of subsequent generations as described previously.^23^, ^24^, ^26^, ^29^, ^31^, ^34^, ^36^, ^38^, ^39^, ^40^, ^41^, ^42^, ^43^ Beyond the 30th generation, GHS rats have consistently excreted 8 to 10 times more urine Ca than of WT SD rats. Urine Ca excretion in SD rats has not varied with time. SD rats purchased from Charles River Laboratories (Kingston, NY, USA) were matched for age and body weight (220 to 250 g) to the GHS rats. All animal experiments were approved by the University of Rochester Animal Care Committee.

### Dual energy x‐ray absorptiometry measurements

As previous studies only assessed BMD in male rats,^36^, ^37^ in this study we also scanned six 3‐month‐old GHS and SD female rats postmortem using a DXA PIXIMus II series densitometer with software version 2 (GE Healthcare, Piscataway, NJ, USA). The scans were carried out following manufacturer's instructions. Because of its larger size, only the posterior part of the rat was scanned. Tibia BMD was measured using the regions of interest tool in the software. Mean values of the two tibia of each of the six animals per group are presented.

### Osteoclasts cultures from bone marrow cells

Bone marrow cells (BMCs) were isolated from femurs of 8‐ to 10‐week‐old male SD and GHS rats. To generate osteoclasts, the isolated BMCs were seeded at a density of 2 × 10^4^ cells/well in a 96‐well plate for tartrate resistant acid phosphatase (TRAcP) staining or 30 × 10^6^ cells/100 mm plate for RNA expression in α‐MEM containing 10% heat‐inactivated FBS, 10 μg/mL penicillin–streptomycin (Hyclone Laboratories, Logan, UT, USA) and 1% L‐glutamine, supplemented with macrophage colony‐stimulating factor (mCSF, 5 ng/mL; R&D Systems, Inc., Minneapolis, MN, USA).^44^, ^45^ After 2‐day incubation, fresh medium containing recombinant soluble RANKL (25 ng/mL; R&D Systems, Inc.) was added to the cells which were cultured for an additional 4 days to induce osteoclast differentiation with an additional medium change after 2 days. The cells in the 96‐well plates were then stained for TRAcP activity; stained cells that contained three or more nuclei, were counted as osteoclasts. The total number of osteoclasts in each well was counted under a microscope (Olympus IX71; Olympus, Waltham, MA, USA) at ×10 magnification. Five wells on each plate and the combined results from three independent experiments are presented. The total area (mm^2^) of osteoclasts in each well was also calculated based on 1 grid square = 0.0045 mm^2^. Total RNA was isolated from the 100‐mm plates using the Rneasy Mini Kit (QIAGEN, Germantown, MD, USA) and used for qPCR analysis of specific osteoclastic gene expression.

### TRAcP assay

Upon completion of the osteoclastogenesis culture, mature osteoclast cells were stained for TRAcP (Sigma‐Aldrich, St. Louis, MO, USA). Dark red TRAcP‐positive cells having three or more nuclei were counted as osteoclasts.

### Resorption pit formation

BMCs were isolated from femurs of 8‐ to 10‐week‐old male SD and GHS rats as above. The isolated cells were seeded at 10^4^ cells/per well on a 50‐μm‐thick bovine cortical bone slice (cut in our laboratory) in a 96‐well plate (one bone slice/per well) and incubated with α‐MEM containing 10% heat‐inactivated FBS and 1% L‐glutamine, supplemented with mCSF (5 ng/mL). After 2 days in culture, the medium was changed to fresh medium also containing soluble RANKL (25 ng/mL). The cells were continually grown on the bone slices with a medium change every 4 days for 9 days. The bone slices were removed from the wells and fixed with 10% neutral buffered formalin for 10 min at room temperature. The cells on the bone slices were removed with a toothbrush and the slices then washed twice with PBS. The bone resorption pit formation was visualized by 100‐μL toluidine blue (1%) staining for 3 min at room temperature. Slices were then rinsed with dH_2_O and air‐dried for imaging. The pit area was quantified using ImageJ software (NIH, Bethesda, MD, USA; https://imagej.nih.gov/ij/).

### Osteoblasts from bone marrow stromal cells

Bone marrow stromal cells (BMSCs) were obtained as described above and plated at 3 × 10^7^ cells/100‐mm plate for RNA collection and at 5 × 10^6^ cells/well in six‐well plates for mineralization. Cells were incubated at 37***°***C in α‐MEM containing 10% FBS, 50‐μg/mL penicillin, and 1% L‐glutamine, supplemented with 50‐μg/mL ascorbic acid. The medium was changed every 3 days. At confluence, 10mM β‐glycerophosphate was also added to the medium, and culture was continued for an additional 21 days, at which time cells were collected for RNA analyses.

### Primary osteoblasts

Calvarial cells were isolated from 2‐ to 3‐day‐old GHS and SD rat pups by sequential collagenase digestion as previously described.^46^, ^47^ The isolated cells were plated in six‐well plates at a density of 5 × 10^5^ cells/well in Dulbecco's modified essential medium. At confluence, fresh α‐MEM, containing 10% heat‐inactivated FBS and 1% L‐glutamine, supplemented with ascorbic acid (50 μg/mL) and 10mM β‐glycerophosphate, was added to the cells to induce cell differentiation and mineralization. The cells were continually grown for another 14 days with a medium change every 3 to 4 days.

### Mineralization assay

After 14 to 21 days in differentiation medium, the cells were rinsed twice with phosphate buffered saline and then fixed in 2 mL of 10% neutral formalin buffer for 15 min at room temperature. After washing with distilled water, the fixed cells were then stained with Alizarin Red solution (1% Alizarin Red S [Sigma] in 95% ethanol) for 30 to 60 min at room temperature. Plates were air‐dried and the relative intensity of positive stain on each well was measured in a ChemiDocMP imaging system (BioRad, Hercules, CA, USA).

### Real‐time PCR

Total RNA was extracted using an RNAeasy minikit (QIAGEN) according to the manufacturer's directions. cDNA synthesis was carried out using an iScript cDNA synthesis kit (BioRad). Quantitative real‐time PCR was performed using SYBR green supermix (Applied Biosystems, Foster City, CA, USA). Quantification of the PCR products was done using the comparative cycle threshold method as described previously.^48^ *Rpl13a* was used as an internal control. Primer sequences are presented in Table 1.

**Table 1:** qPCR Primer Sequences

Primer	Forward	Reverse
*Tracp*	5′‐CAGCAGCCAAGGAGGACTAT‐3′	5′‐ACATAGCCCACACCGTTCTC‐3′
*Ctsk*	5′‐AGACGCTTACCCGTATGTGG‐3′	5′‐GGACACAGAGACGGGTCCTA‐3′
*Rank*	5′‐CTGCTCCTCTTCATCTCTGTG‐3′	5′‐CTTCTGGAACCATCTTCTCCTC‐3′
*Csf1r*	5′‐GCCTTTGGTCTGGGCAAA‐3′	5′‐AGCCGTGGACTTGAGCATCT‐3′
*Nfatc1*	5′‐CAACGCCCTGACCACCGATAG‐3′	5′‐GGCTGCCTTCCGTCTCATAGT‐3′
*Mmp9*	5′‐TCGAAGGCGACCTCAAGTG‐3′	5′‐TTCGGTGTAGCTTTGGATCCA‐3′
*Alp*	5′‐GGACGGTGAACGGGAGAAC‐3′	5′‐TGAAGCAGGTGAGCCATAGG‐3′
*Casr*	5′‐TTCTATCATCAACTGGCACCTC‐3′	5′‐TTGTCACAGGCACTCGCATCTG‐3′
*Bmp*	5′‐GAAGCCAGGTGTCTCCAAGAG‐3′	5′‐GTGGATGTCCTTTACCGTCGT‐3′
*Opg*	5′‐GTCCCTTGCCCTGACTACTCT‐3′	5′‐GACATCTTTTGCAAACCGTGT‐3′
*Rankl*	5′‐GGGCCAAGATCTCTAACATGA‐3′	5′‐TCATGATGCCTGAAGCAAATG‐3′
*Col1*	5′‐GAGTGGGGAACACACAGGTCT‐3′	5′‐TCTGACTGGAAGAGCGGAGAG‐3′
*Runx2*	5′‐GCCGGGAATGATGAGAACTA‐3′	5′‐GGACCGTCCACTGTCACTTT‐3′
*Spp1*	5′‐CTGCCAGCACACAAGCAGAC‐3′	5′‐TCTGTGGCATCGGGATACTG‐3′
*Ocn*	5′‐CACCTTACTGCCCTCCTGCTT‐3′	5′‐GACCCTCTCTCTGCTCACTCTG‐3′
*Ostx*	5′‐AACAGCCCTGGGAAAAGGAGGC‐3′	5′‐GGAGTCCATTGGTCGTTGAGA‐3′
*Rpl13a*	5′‐GGATCCCTCCACCCTATGACA‐3′	5′‐CTGGTACTTCCACCCGACCTC‐3′

### Data analysis

Statistical analysis was performed using the Student's *t* test for unpaired comparisons. Data were presented as means ± SEM; *p* < 0.05 was considered significant.

## Results

### Bone mineral density

**Figure 1:**
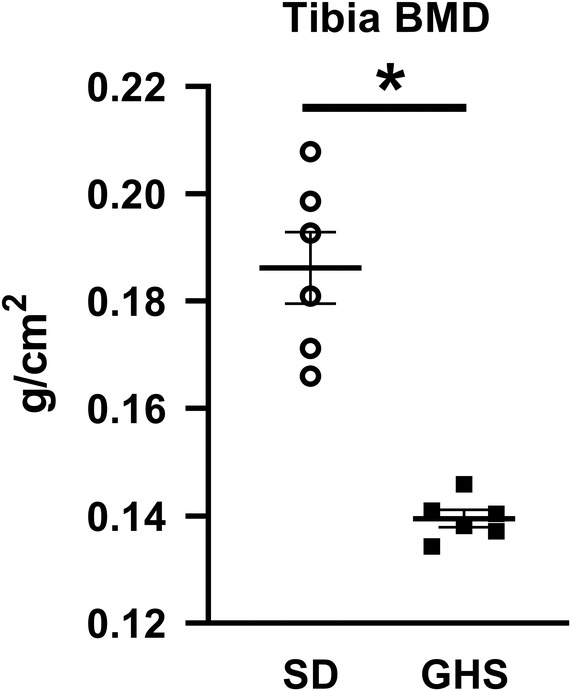
Decreased BMD in genetic hypercalciuric stone‐forming (GHS) female rats. Tibias from Sprague–Dawley (SD) and GHS female rats were scanned postmortem. The average BMD of both tibias/rat is presented. Results are mean ± SE for six rats/group. *p < 0.05 compared with SD rats.

As we have found previously in male rats,^36^, ^37^ the BMD of female rat tibias measured by DXA was lower in GHS rats compared with SD controls (Fig. 1), indicating that the hypercalciuria and subsequent decrease in BMD is not a sex‐linked trait.

### Osteoclasts

**Figure 2:**
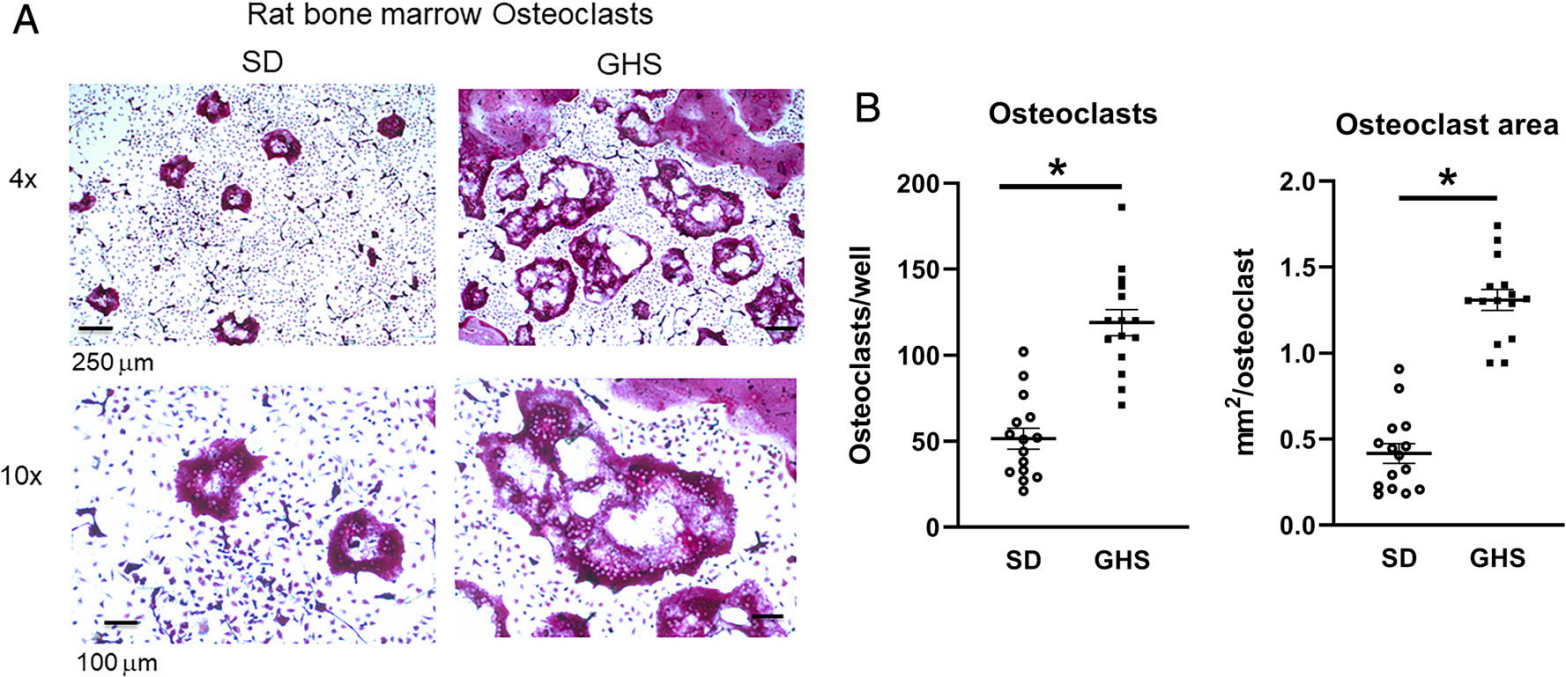
Increased osteoclasts in cells differentiated from genetic hypercalciuric stone‐forming (GHS) rat bone marrow. Rat bone marrow from 3‐month‐old male rats was differentiated to osteoclasts as described in the Materials and Methods section. After TRAcP (tartrate resistant acid phosphatase) staining, cells in the 96‐well plates were counted. (*A*) Micrograph of representative wells from Sprague–Dawley (SD) and GHS cells at ×4 and ×10 magnification. (*B*) Quantitation of number of osteoclasts/well (left panel) and of osteoclast area/well (right panel). Results are mean ± SE for 15 wells/group from three separate experiments. *p < 0.05 compared with osteoclasts from SD rats.

**Figure 3:**
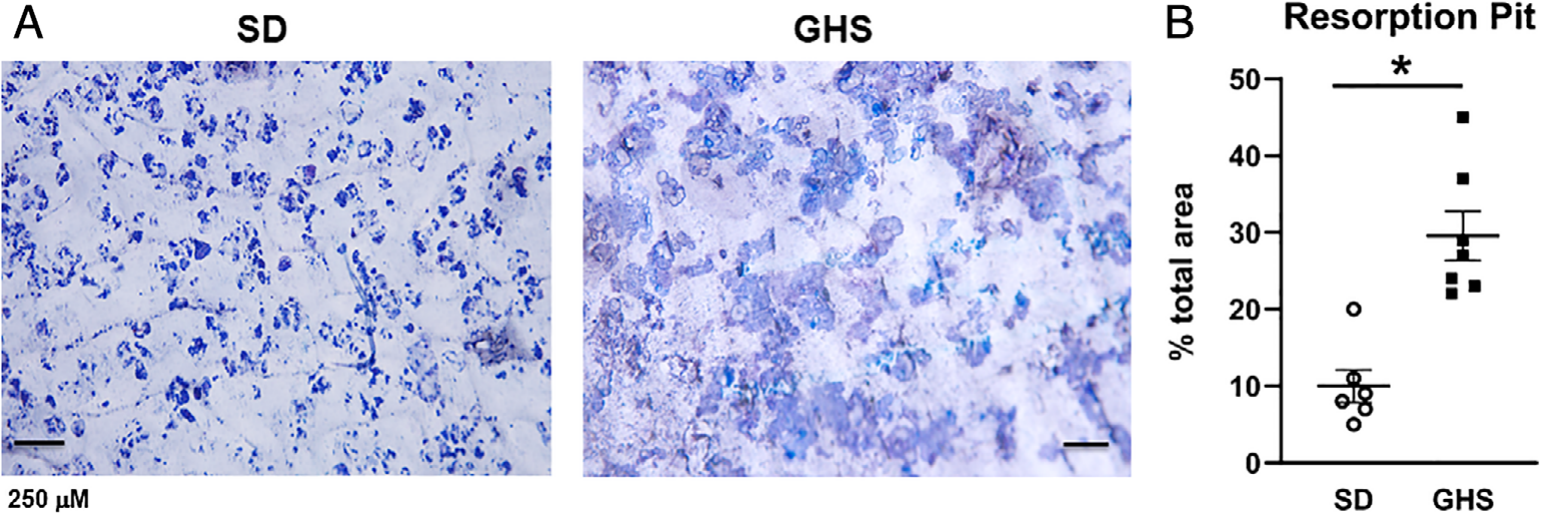
Increased resorption pit formation on bone slices in the presence of differentiated bone marrow cells (BMCs) from genetic hypercalciuric stone‐forming (GHS) rats. BMCs from Sprague–Dawley (SD) and GHS rats were plated on bovine cortical bone slices in 96‐well plates and differentiated to osteoclasts. (*A*) Cells were then removed and the slices were stained with toluidine blue. (*B*) Quantitation of the resorption pit area. Results are mean ± SE for five slices/group. *p < 0.05 compared with bones plated with SD cells.

To understand whether osteoclast or osteoblast activity is altered in these rats, leading to the changes in BMD, we examined isolated cell cultures. GHS rat BMCs cultured for 6 days with mCSF and RANKL developed increased numbers of mature osteoclasts compared with SD rats (Fig. 2 *A*). The number of the TRAcP‐positive multinucleated cells and the size of the osteoclasts were greater from cells isolated from GHS rats than those from SD rats (Fig. 2 *B*). When these differentiated osteoclasts were cultured on bovine bone slices, osteoclasts from GHS rats created more resorption pits (Fig. 3 *A*) with greater area of erosion compared with SD rat‐derived cells (Fig. 3 *B*).

**Figure 4:**
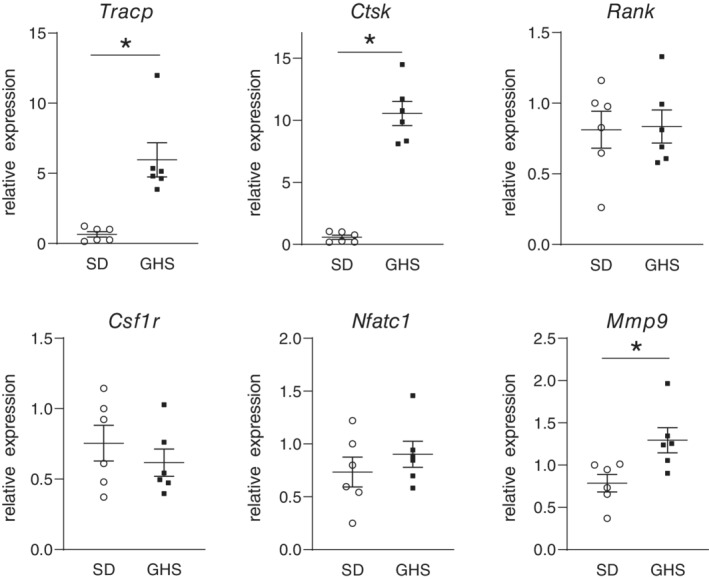
Changes in gene expression in differentiated osteoclasts from genetic hypercalciuric stone‐forming (GHS) and Sprague–Dawley (SD) rats. Bone marrow cells were isolated from 3‐month‐old SD and GHS rats and differentiated to osteoclasts as described in the Materials and Methods section. RNA was collected from cells and used for qPCR. Results are mean ± SE for the relative expression of tartrate resistant acid phosphatase (*Tracp*), cathepsin K (*Ctsk*), receptor activator of NfKappaB (*Rank*), colony stimulating factor‐receptor 1 (*Csfr1*), nuclear factor of activated *T* cells 1 (*Nfatc1*), and matrix metallopeptidase 9 (*Mmp9*). Open circles = SD‐derived osteoclasts; closed squares = GHS‐derived osteoclasts. *p < 0.05 compared with SD expression.

The marrow cells from GHS and SD rats, cultured under conditions that led to differentiated osteoclasts, were also collected, and RNA prepared for analysis of specific osteoclastic gene expression. There was a significant increase in *Tracp*, *cathepsin K*, and *Mmp9* expression in GHS compared with SD osteoclasts (Fig. 4).

### Osteoblasts

**Figure 5:**
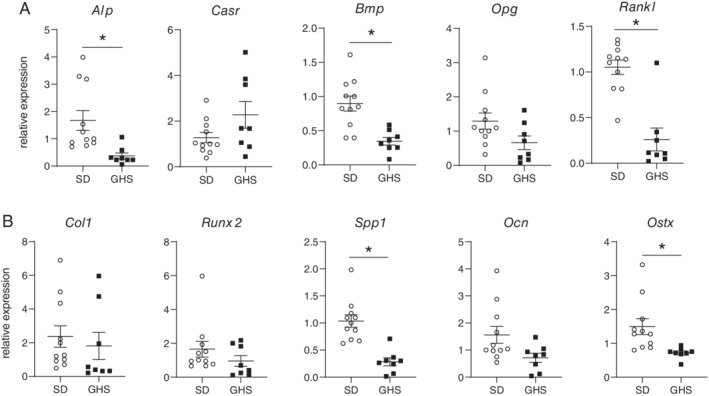
Decreased gene expression in osteoblasts differentiated from bone marrow stromal cells (BMSCs) from genetic hypercalciuric stone‐forming (GHS) rats compared with Sprague–Dawley (SD) rats. BMSCs from SD and GHS rats were differentiated to osteoblasts. After 3 weeks in differentiation medium, RNA was collected from cells and used for qPCR. Results are mean ± SE for 11 to 14 samples/group. (*A*) Relative expression of alkaline phosphatase (*Alp*), Ca sensing receptor (*Casr*), bone morphogenic protein (*Bmp*), osteoprotegerin (*Opg*), and receptor activator of NfKappaB ligand (*Rankl*). (*B*) Relative expression of collagen1a1 (*Col1*), (*Runx2)*, osteopontin (*Spp1*), osteocalcin (*Ocn*), and osterix (*Ostx*) are presented. Open circles = SD derived osteoblasts; closed squares = GHS‐derived osteoblasts. *p < 0.05 compared with SD expression.

**Figure 6:**
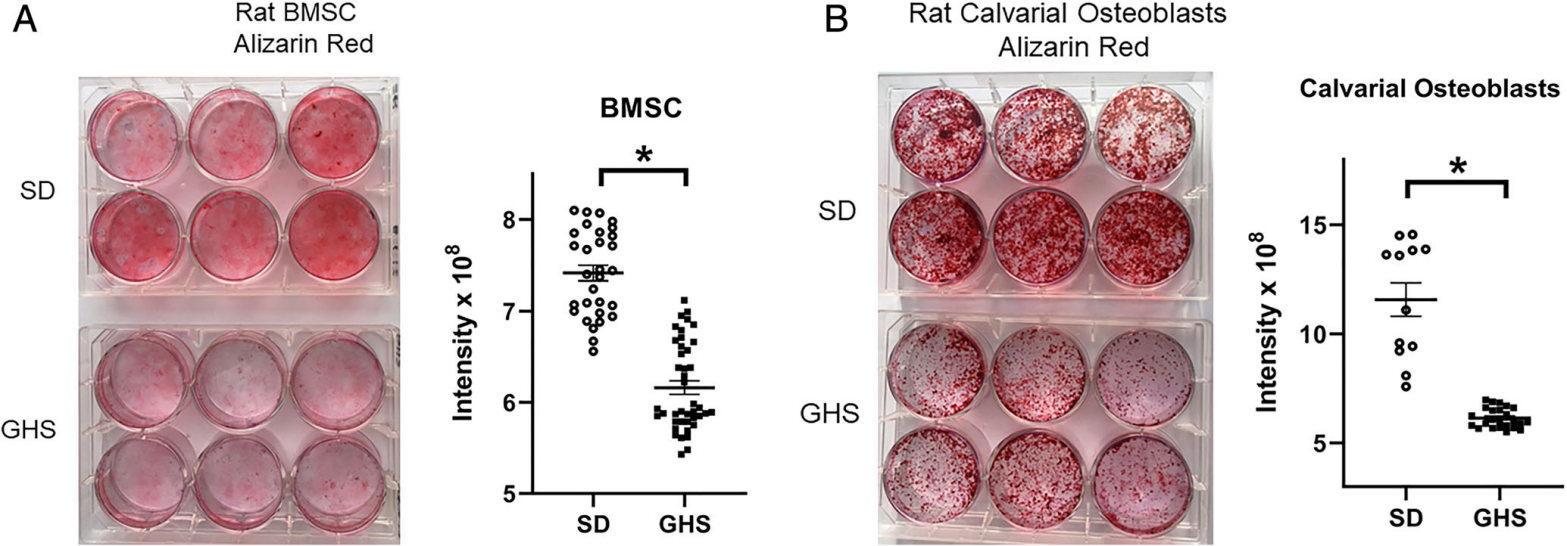
Decreased mineralization from osteoblasts differentiated from bone marrow stromal cells (BMSCs) and neonatal rat calvariae from genetic hypercalciuric stone‐forming (GHS) rats. Cells were isolated from Sprague–Dawley (SD) and GHS femurs or neonatal rat pups and differentiated to mature osteoblasts as described in the Materials and Methods section. After 14 (calvarial cells) or 21 (BMSCs) days in differentiation medium, cells were stained with Alizarin Red. (*A*) Mineralizing osteoblasts differentiated from BMSCs (left panel) and quantitated for intensity of staining (right panel). (*B*) Mineralizing osteoblasts differentiated from neonatal calvarial cells (left panel) and quantitated for intensity of staining (right panel). Results are mean ± SE. *p < 0.05 versus SD cells.

Specific osteoblast responses were also characterized. BMSCs were obtained from GHS and SD rat femurs and differentiated to osteoblasts. After 3 weeks in differentiation medium, cells were collected and RNA was isolated for qPCR. The relative expression of osteoblastic genes is shown in Fig. 5 *A,B*. Significant decreases were observed in the expression of alkaline phosphatase, *Bmp*, *Rankl*, osteopontin, and osterix in GHS osteoblasts compared with SD osteoblasts. To determine changes in mineralization of these cells, other differentiated marrow cells were collected and stained with Alizarin Red. Differentiated osteoblasts from GHS BMSCs exhibited significantly less mineralization compared with cells from SD rats (Fig. 6 *A*). Similar results were obtained when mature calvarial osteoblasts were cultured in differentiation medium (Fig. 6 *B*).

## Discussion

Calcium nephrolithiasis and low bone mass leading to fracture are important clinical manifestations of human IH.^7^, ^8^, ^9^, ^10^, ^11^ The pathogenesis of reduced bone mass in IH remains incompletely understood in part because of the wide variability in the genetic background across human populations and their variable Ca, sodium, protein intake, and vitamin D status. Using the GHS rats, which share important physiological parallels to human IH, have little genetic variability, and are fed a similar diet, we^36^ demonstrated primary abnormalities in bone.^36^ We found decreased cortical bone density and reduced trabecular bone volume and thickness that were not corrected by a high Ca diet and were worsened with a low Ca diet. Administration of the long‐acting thiazide diuretic agent chlorthalidone (CTD) reduced urine Ca excretion in GHS rats and improved trabecular volume, number, thickness, and mineralization. As a result, vertebral strength and stiffness improved.^38^ The reversal of hypercalciuria by chlorthalidone administration improves BMD and bone quality toward normal.^38^ The results strongly suggest that low bone mass and trabecular and cortical defects in GHS rats are causally linked to the high urine Ca excretion. Similar improvement in BMD and risk of hip fracture has been reported in IH subjects and others whose urine Ca had been lowered by treatment with thiazide diuretics.^49^, ^50^, ^51^, ^52^ We have shown that the hypercalciuria in the rat model is a polygenic trait^53^, ^54^ and there is an increase in vitamin D receptors (VDRs) in bone, intestine, and kidney in the GHS rats compared with SD rats^22^ that contributes significantly to Ca mobilization in these rats.

In the present study, as we have seen previously with male rats,^36^ female GHS rats had lower BMD compared with SD rats, indicating that the reduction of BMD does not appear to be a sex‐linked trait. When primary cultures of BMCs were differentiated to osteoclasts, cells from GHS rats showed increased osteoclastic resorption activity compared with osteoclasts from SD rats. The in vitro addition of RANKL stimulated differentiation of GHS rat BMCs into mature osteoclasts, which were greater in individual size, number, and number of nuclei than those from SD rats. We also found an increase in *Tracp*, *cathepsin K*, and *Mmp9* gene expression, three markers of mature osteoclasts, in osteoclasts differentiated from marrow cells of GHS rats compared with cells from SD rats. There were no significant changes in *Rank, Csf1r*, or *Nfatc1* expression. It is not clear why there was no significant increase in *Nfatc1*, though there were increases in its downstream genes, such as *Ctsk*. In our experiments there was a trend to increased expression of *Nfatc1* in the GHS osteoclasts, so a larger sample size might have led to a significant increase. These results provide a mechanism for increased bone resorption leading to decreased BMD in the GHS rats.

When BMSCs were differentiated to osteoblasts, we also found that cells from GHS rats demonstrated decreased expression of multiple osteoblastic genes compared with the gene expression of cells from SD rats. Furthermore, mineralization of differentiated osteoblasts derived from BMSCs as well as calvarial osteoblasts was decreased in GHS cells compared with SD cells. Both of these results suggest a mechanism for decreased bone formation in the GHS rats. The decrease in *Rankl* expression is not entirely expected given our finding of increased osteoclast differentiation. However, RANKL‐independent stimulation of osteoclasts has been described,^55^ so in future experiments we will also examine whether there are significant changes in other cytokines, such as TNFα, that could account for the stimulation of osteoclastic bone resorption in these rats.

In a previous study using neonatal GHS rat calvariae, we found that 1,25(OH)_2_‐vitamin D_3_ directly stimulated a greater rise in resorption by GHS rat bone compared with control rats at the same dose of 1,25(OH)_2_‐vitamin D_3._ 26 The increased sensitivity of GHS bone to in vitro 1,25(OH)_2_‐vitamin D_3_ and the hyper‐responsiveness to in vitro RANKL in the present study suggest that the high VDR levels in GHS rat BMSCs could enhance RANKL overexpression and osteoclastic bone resorption. The present study supports RANKL as an important mediator of bone resorption in GHS rats. PTH is an unlikely mediator of increased bone resorption in GHS rats as PTH levels are not elevated in GHS rats, and in vitro addition of PTH caused comparable stimulation of bone resorption in GHS and SD rat calvariae.^26^ Future studies will determine whether the VDR—1,25‐dihydroxyvitamin D_3_ complex may increase osteoclastic bone resorption in GHS rat bone through upregulation of osteoclastic RANKL gene expression. Defects in genes that determine peak skeletal mass were not examined in this study; however, the possibility remains that lower skeletal mass in GHS rats is caused by lifelong alterations in osteoclast activity.

The present study does not address the time of appearance of the increased bone resorption in the GHS rats; however, the hypercalciuria is present as early as 6 weeks of life upon completion of weaning, and the hyper‐response of bone resorption to 1,25(OH)_2_D_3_ and the high bone cell VDR content are present in calvariae of 2‐day‐old pups from GHS mothers.^26^ Thus, the functional changes in GHS bone cells may be lifelong.

We conclude from the present study in GHS rats that increased osteoclastic differentiation and function with concomitant decreased osteoblastic activity lead to the decreased bone density and changes in bone quality in these hypercalciuric, stone‐forming rats. If comparable changes in bone cell function are present in humans with IH, these changes could contribute to their bone fragility and increased rate of fracture, and may provide insight and lead to strategies to mitigate their low BMD and increased fracture risk.

## Disclosures

DAB reports consulting fees from Sanofi/Genzyme, Relypsa/Vifor/Fresenius, OPKO, Tricida, and Sanifit and owns stock in Amgen Inc. and stock and stock options in Tricida: All are not related to this work. All other authors have nothing to disclose.
